# Cyberinfrastructure for the digital brain: spatial standards for integrating rodent brain atlases

**DOI:** 10.3389/fninf.2014.00074

**Published:** 2014-09-12

**Authors:** Ilya Zaslavsky, Richard A. Baldock, Jyl Boline

**Affiliations:** ^1^San Diego Supercomputer Center, University of California San DiegoLa Jolla, CA, USA; ^2^MRC Human Genetics Unit, Institute of Genetics and Molecular Medicine, University of EdinburghEdinburgh, UK; ^3^Informed Minds, Wilton ManorsFL, USA

**Keywords:** digital atlases, atlas infrastructure, spatial data integration, brain coordinate systems, Waxholm space, atlas services, coordinate transformations

## Abstract

Biomedical research entails capture and analysis of massive data volumes and new discoveries arise from data-integration and mining. This is only possible if data can be mapped onto a common framework such as the genome for genomic data. In neuroscience, the framework is intrinsically spatial and based on a number of paper atlases. This cannot meet today's data-intensive analysis and integration challenges. A scalable and extensible software infrastructure that is standards based but open for novel data and resources, is required for integrating information such as signal distributions, gene-expression, neuronal connectivity, electrophysiology, anatomy, and developmental processes. Therefore, the International Neuroinformatics Coordinating Facility (INCF) initiated the development of a spatial framework for neuroscience data integration with an associated Digital Atlasing Infrastructure (DAI). A prototype implementation of this infrastructure for the rodent brain is reported here. The infrastructure is based on a collection of reference spaces to which data is mapped at the required resolution, such as the Waxholm Space (WHS), a 3D reconstruction of the brain generated using high-resolution, multi-channel microMRI. The core standards of the digital atlasing service-oriented infrastructure include Waxholm Markup Language (WaxML): XML schema expressing a uniform information model for key elements such as coordinate systems, transformations, points of interest (POI)s, labels, and annotations; and Atlas Web Services: interfaces for querying and updating atlas data. The services return WaxML-encoded documents with information about capabilities, spatial reference systems (SRSs) and structures, and execute coordinate transformations and POI-based requests. Key elements of INCF-DAI cyberinfrastructure have been prototyped for both mouse and rat brain atlas sources, including the Allen Mouse Brain Atlas, UCSD Cell-Centered Database, and Edinburgh Mouse Atlas Project.

## Introduction

Frequently asked questions in neuroscience are “where” in the brain something is happening, “what” is happening “here,” and “what” is this structure. The extended version asks for similarity and association between biological processes and structures to understand complex observations. Most researchers, in one way or another, access information from a reference brain atlas and apply the associated material to their own datasets. This allows them to compare and analyze data within their own laboratories as well as in relation to outside sources. Mouse brain atlases were initially developed as paper atlases (Hof et al., 2000; Paxinos, 2004; Paxinos et al., 2007; Paxinos and Watson, 2009), and have been used in this form for many years to support spatial referencing in electrophysiology and other studies. Recently, atlas providers have put significant effort into organizing atlas information in digital form, creating digital brain atlases as collections of spatially and semantically consistent 2D images or 3D volumes with anatomical structure delineations and additional annotations. These atlases have been made accessible via desktop [e.g., MRM NeAT (http://brainatlas.mbi.ufl.edu/), Mouse Atlas Project (http://map.loni.usc.edu/), CIVM (http://www.civm.duhs.duke.edu/)] and online interfaces such as the Allen Brain Atlas (http://www.brain-map.org/), EMAP, (http://www.emouseatlas.org/emap/home.html), MBL (http://www.mbl.org/mbl_main/atlas.html) Mouse Brain Atlas http://www.hms.harvard.edu/research/brain/atlas.html, Genepaint (http://genepaint.org/Frameset.html), Australian Mouse Brain Mapping Consortium (http://www.tissuestack.org), Rodent Brain WorkBench (http://www.rbwb.org/), Laboratory of Brain Anatomical MRI (http://lbam.med.jhmi.edu/), Knife-Edge Scanning Microscope Brain Atlas (http://kesm.cs.tamu.edu/), and SumsDB (http://sumsdb.wustl.edu/).

While such atlases have been internally consistent, they have been developed largely independently of one another. Without uniform conventions for brain data representation and access, users have limited ability to quickly answer questions such as “which atlas-based resources have images for a specified part of the brain,” “what genes are expressed in a given tissue in atlases A and B, at a specified expression level,” “compare spatial patterns of protein distribution across atlases C and D,” or “what proteins are expressed in the projection domains of hippocampal neurons.” Yet answering such questions becomes increasingly important in neuroscience and other domains as scientists attempt to integrate information and knowledge encapsulated in multiple information sources to test hypotheses or to infer novel associations and patterns in an atlasing environment (Bjaalie, 2002; Toga, 2002; Baldock et al., 2003; MacKenzie-Graham et al., 2003; Martone et al., 2004; Zaslavsky et al., 2004; Boline et al., 2008; Hawrylycz et al., 2011; Zakiewicz et al., 2011).

While this type of environment has been desired by many members of the neuroscience community for quite some time now, a spatial framework that enables interoperability between existing atlasing efforts and allows the addition of other spatially-tied data has not been built for technical, social, and financial reasons. Creating such an environment has been one of the foremost goals of the Digital Atlasing Program of the International Neuroinformatics Coordination Facility, INCF (Hawrylycz et al., 2009, 2011). Under this program, INCF has brought together a group of neuroscientists and technology experts to organize atlas resources, explore and outline best practices and recommendations, and design and guide the development of standards, information infrastructure, and tools for integrating digital brain atlases.

Use cases established over recent years^1^ show that most neuroscientists want to have the ability to bring together and compare different types of information: explore a reference atlas, juxtapose it with their own data, and finally, link and compare their data to other datasets. For instance, researchers using immunohistochemistry to examine images for a specific protein may not have much anatomical information in the images. Applying atlas delineations from a canonical atlas to their images would let them examine and quantify the level of labeling in different brain areas. With this information, they may wish to run a quantitative analysis that compares their data to another resource, such as the Allen Brain Atlas and then visualize it in 3D.

**Figure 1:**
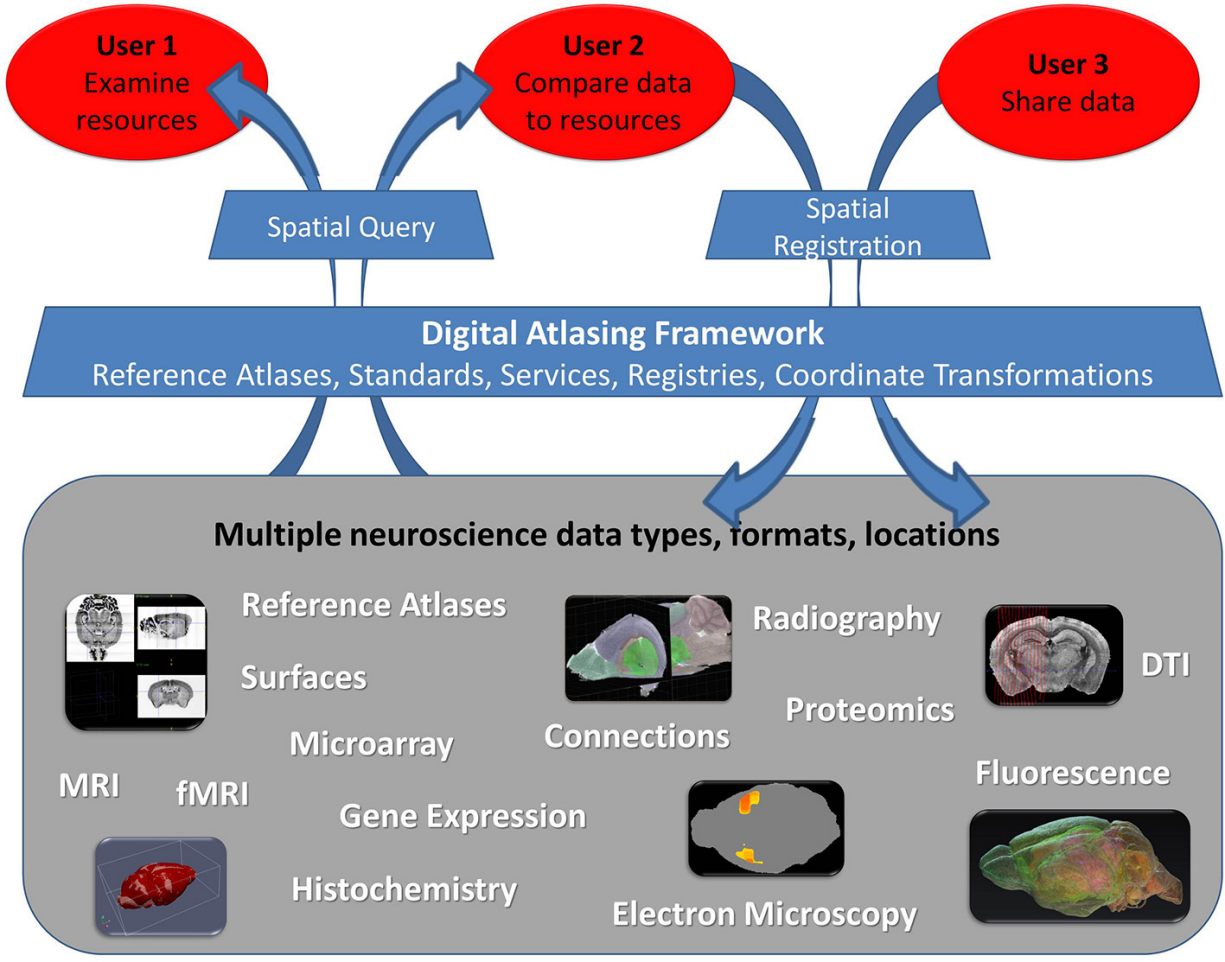
**Three user groups interacting with neuroscience data within the digital atlas framework**. The framework should allow integration of datasets of various type, format, and location through the Digital Atlasing Infrastructure (DAI). Users are able to interact with this environment using DAI tools, which enable spatial query of data shared through this framework or addition of new data via spatial registration. Note that we differentiate data sharing mechanisms for User 2 and User 3: User 2 typically has a limited number of images and needs to register them primarily to explore other atlas sources spatially, while User 3 typically shares large volumes of spatially-referenced data within their group or to others, for the purpose of making it available for query and more automated analyses in a spatial framework. User 3 may even have their own reference atlas. The framework can be expanded to accommodate additional data types beyond those shown.

The compendium of use cases allowed us to identify three groups of researchers based on their use of atlases (Figure 1). The most basic need is simply to find and examine information about their area of interest (Figure 1, User 1). Another group wants capabilities that include relating user resources with external canonical atlases based on spatial properties, such as location, shape or observed spatial pattern (Figure 1, User 2). Finally a number of users want to share their data with others such that image collections, 3D reconstructions, gene expression or other information they collected can be accessed online and used as a reference in a given spatial framework (Figure 1, User 3). While simply posting data online is possible, placing the information into a known spatial framework provides the ability to run novel analyses (Carson et al., 2005; Kovacević et al., 2005; Christiansen et al., 2006; Leergaard and Bjaalie, 2007; Lein et al., 2007; Ma et al., 2008; Aggarwal et al., 2009; Ng et al., 2009; Chuang et al., 2011) and to integrate data from different atlas-based resources (Baldock et al., 2003; MacKenzie-Graham et al., 2004; Martone et al., 2004; Boline et al., 2008; Lee et al., 2010; Hawrylycz et al., 2011). Most users want to do this at some point, but many have no idea how to even start the process. This is an extremely daunting task, due, to a large degree, to the complete lack or complexity of sharing conventions for atlas data and supporting data publication tools. Meeting the needs of all these users through the creation of a flexible, expandable, and accessible spatial framework for sharing atlas data has been one of the main goals of the INCF Digital Atlasing Program.

A key component of this open framework is a common publicly accessible 3D reference space, providing standard coordinates and serving as a spatial anchor for other existing rodent brain atlas resources (Hawrylycz et al., 2011). Such a canonical Waxholm Space (WHS) has been developed for C57BL/6J mouse (Johnson et al., 2010). In addition, two recent versions of WHS for the rat, one Sprague Dawley (Johnson et al., 2012) and one Wistar (Papp et al., 2013) have been created. The goal is to embed them as the rat spatial anchors of our framework, register them to each other and to create a mapping from mouse to rat. In addition to standardizing reference spaces, agreements about how location information is represented and exchanged between atlases must be established—these agreements are the foundation of software infrastructure that support publication, discovery, access, and integration of distributed atlas information.

We have developed the underlying principles and implemented a prototype of an open standards-based spatial data integration framework, the Digital Atlasing Infrastructure (DAI). This includes the backbone of the infrastructure itself, along with a few online applications and tutorials to enable neuroscientists to use and add to the infrastructure. We expect that a rich set of supporting tools will be developed over time by members of the neuroscience and neuroinformatics communities leveraging standards-based information exchange protocols tested in the prototype.

This article describes the DAI, including its rationale, components, and the current state of the system. We focus on the formal definition of coordinate systems and coordinate transformations for rodent brain, a service interface for DAI services, and a standards-based XML schema for encoding atlas information, called Waxholm^2^ Markup Language (WaxML). It is followed by implementation details, and a description of a spatial registration pipeline, which illustrates how to extend the system with additional spatially-referenced data. Finally, we address the benefits of leveraging existing spatial integration frameworks and standards for atlas data integration, and future work.

## Digital atlasing infrastructure: high-level requirements and main components

The vision of brain atlases as interconnected gateways to large distributed and diverse atlas resources, including images, volume data, segmentations, gene expression, electrophysiology, behavioral, connectivity, other spatially-organized data, implies a number of design requirements:

- Atlases should be organized as spatial data sources, which support querying atlas data using spatial characteristics of their content, in particular by coordinates in a brain coordinate system.
- Information from multiple brain atlas sources should be available for searching and browsing, which typically involves indexing data elements in a spatial data registry.
- The spatial data and metadata must be accessible via standard protocols and in common formats, following established standard application programming interfaces (APIs) and information models. In addition, capabilities of each atlas resource should be advertised in a standard manner, so that different functions can be automatically invoked and chained to implement data integration and research workflows.
- DAI should incorporate transparent and easy to follow mechanisms for users to extend the system: by publishing and registering spatially-referenced atlas data, via standards-compliant spatial registration pipelines, and through annotation or segmentation.
- Brain atlas data must be accessible to a number of desktop and web-based data management, cataloging, analysis, visualization, and other applications that take advantage of the uniform APIs and information encodings. This model allows software developers the ability to use this resource for very different application needs.
- Ideally, most of the underlying services infrastructure will be invisible to the neuroscientists working through easy to use software tools that directly access DAI via standard APIs. As user needs evolve and the complexity of sharing or accessing data in a spatial framework increases, DAI will need continuing participation of neuroscience researchers to guide infrastructure development, through the INCF Digital Atlasing Program or similar mechanisms.

**Figure 2:**
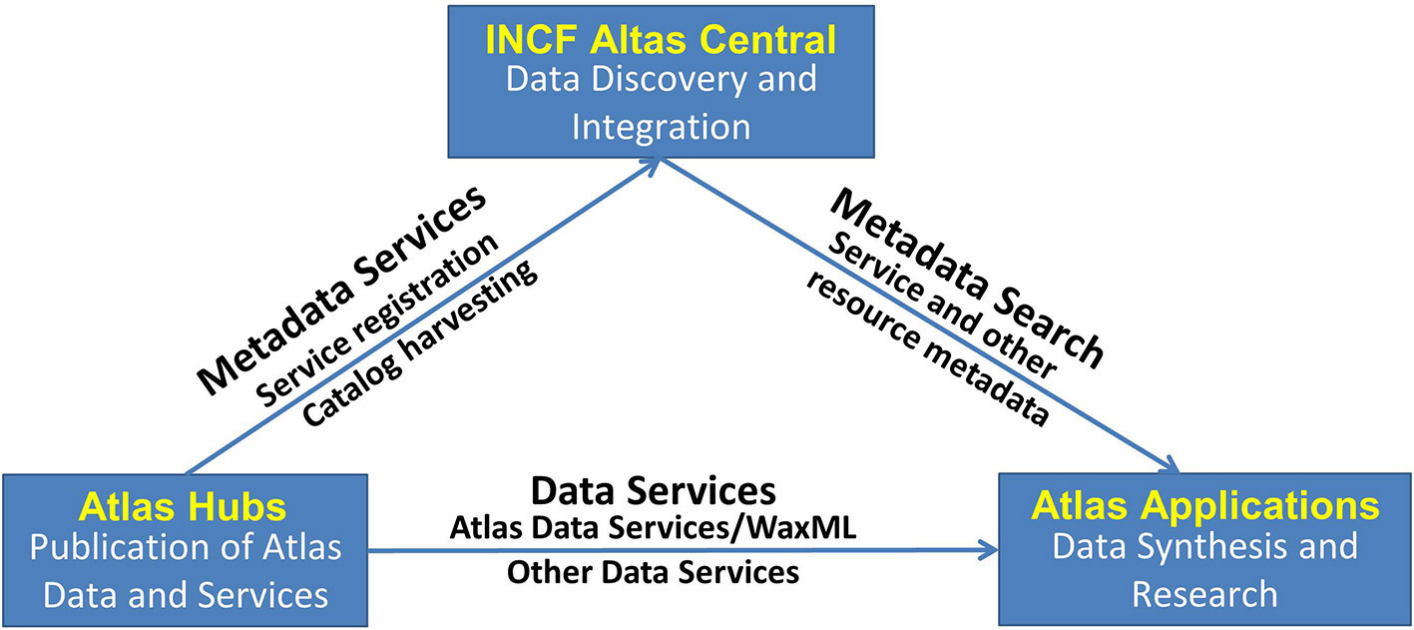
**High-level design of the INCF Digital Atlasing Infrastructure**. The design follows the standard SOA “publish-find-bind” pattern, bringing together providers of atlas data and services, catalog and discovery services, and data synthesis and research applications. *Atlas Hubs* share their data via DAI-compatible services. *INCF Atlas Central* contains a catalog of what is available from the Atlas Hubs and also acts as a “translator” between the different spatial coordinates offered by the Atlas Hubs. Various *Applications* can be developed that use INCF Atlas Central to find what is available and then access the services offered by the Atlas Hubs. This SOA-based design allows significant flexibility in tool development.

The DAI follows service-oriented architecture (SOA) principles (Erl, 2005; Josuttis, 2007), whereby atlas information becomes available via *atlas web services*, a collection of functions that deliver spatial and other information in standardized agreed-upon formats, thus alleviating the existing heterogeneity across different atlas resources. The high-level system architecture includes three key logical components (Figure 2):

- Atlas Hubs—an atlas data publication platform: a software stack for publishing neuroscience atlas data and web services, compliant with the WaxML schema and atlas services specification. An atlas hub may be maintained by an atlas-related project, or hosted by INCF as a proxy of a remote atlas resource.
- INCF Atlas Central—the central data discovery and integration platform: a catalog of atlas web services from multiple hubs, as well as other atlas-related data. Using standard catalog services, users and applications can search for appropriate web services across atlas hubs. In addition, the INCF Atlas Central system contains a special “central atlas hub” designed as a mediator for coordinate transformation services invoked across multiple hubs.
- Atlas Applications—the data synthesis and research platform: a collection of analysis, visualization, modeling, and other applications that consume standard atlas data and metadata (catalog) services, or are used to manage and update atlas information at a hub. Such applications include, for example, the INCF Scalable Brain Atlas and the UCSD Web Image Browser (WIB), developed by different DAI partners.

The initial focus of the atlasing infrastructure is limited to representation of anatomic features in the brain, brain reference systems and coordinate transformations, fiducial points and landmarks, and a few types of spatially referenced data and annotations that can be retrieved using point of interest (POI) requests. These functions fit the needs of our “User 1,” those looking for spatially-linked data. In our review of existing online atlases of rodent brain we found significant heterogeneity in modalities, formats and functionality. Individual atlas resources may support different data types and use different metadata and data representations; they have been developed using different data collection methods; support different data retrieval, processing and other functions, and often adhere to different spatial and semantic frameworks. For example, a neuroscientist might want to use POI requests to find the name of the structure at this POI in WHS, the Allen Mouse Brain Atlas, or a Paxinos annotated atlas. They may wish to discover all available images in the vicinity of the POI regardless of atlases that contain them. However, some existing atlas resources may not support structure or image retrieval based on brain location; the structure names often belong to different vocabularies; and structure geometries depend on different delineation techniques, complicating cross-comparison. Similarly, any discovered images are likely to be in different formats and reflect different measurement modalities and instruments.

This heterogeneity presents an informatics challenge in developing an interoperable system for brain information that can work across multiple, independently managed, atlas information sources, processing services, and client applications. Hence, development of shared information models and data exchange protocols, and information brokers, is a central requirement for designing communication across DAI components. Establishing community consensus about information models and exchange protocols ensures that infrastructure components are structurally interoperable. Standards-compliance also enhances extensibility of the atlas infrastructure, by making it easier to incorporate standards-based software modules created by developers outside the DAI project. Consequently, maintenance of standards-based systems is usually less expensive, and expertise is easier to find because it does not have to come from a single group. In the long run, such systems evolve more easily with changes in technology, and are more economical as they encourage cooperation, competition, and prevent a software vendor lock-in (David and Greenstein, 1990; West, 2007).

Development of consensus about data sharing formats and protocols, and their community adoption, is a long process; therefore, one of the key requirements of the DAI is enabling evolution of the system to such standard conventions rather than enforcing rigid standards compliance from the start. As described in the next section, this approach is adopted in the choices for specifying and implementing atlas services, markup, and in defining spatial reference systems (SRSs) and transformations.

## Standardization of spatial representation and spatial data access to rodent brain data

Three standard components need to be specified in an interoperable atlas infrastructure design: (1) a common spatial framework, (2) the structure of key information elements to be exchanged across atlases, and (3) the respective exchange protocols.

### Common spatial framework

Established paper atlases of rodent brain (Paxinos and Watson, 1998; Swanson, 1998; Hof et al., 2000; Paxinos and Franklin, 2001) include coordinate systems used to describe anatomic feature locations and relationships in terms of distance to key brain landmarks (e.g., bregma, midline) and neuroscience anatomical axes: dorsal-ventral, anterior-posterior, left-right. In some cases, such feature-based coordinate systems are combined with image-based coordinates, but most typically, for a collection of images forming an atlas, locations are only referenced by a slice index and by image coordinates within the slice. Due to a wide variety of imaging and processing techniques, and different physical properties of the sectioned brains, there is little consistency across such spatial descriptions, which makes it difficult to translate location information from one atlas to another and subsequently integrate data based on location in the brain except in the most cursory manner.

A similar problem has been recognized and resolved in geodesy, where many coordinate systems have been developed over the centuries for different purposes, at different resolutions, using different models of the earth, and allowing for different types of distortions (in direction, area, shape, distance). The solution involved several components:

- development of more accurate mathematical descriptions of the shape of the earth;
- creating precise and consistent models of projections as transformations from earth coordinates into various 2D and 3D digital representations;
- standardization of coordinate transformation descriptions (e.g., the OpenGIS Coordinate Transformation Service Implementation Specification, see http://www.opengeospatial.org/standards/ct);
- cataloging the available coordinate systems (e.g., the EPSG Geodetic Parameter Dataset); and
- development of widely used coordinate transformation packages (e.g., the General Cartographic Transformation Package).

Registries of coordinate systems and coordinate transformation libraries are foundational components of global spatial data infrastructure; they are accessed from multiple spatial information system software packages. For example, the geospatial SRS registry (http://www.epsg-registry.org/) contains definitions of thousands of SRSs. For each system, the description includes a code (e.g., EPSG:4326), which is used by process libraries, web services and other software applications to reference the SRS; name (e.g., World Geodetic System 1984 or WGS84), type of SRS (e.g., “geographic 2D”), specification of the “Area of Use” (e.g., “world”), as well as description of the underlying geodetic datum, projection conversion, and versions/revisions.

While definitions of brain coordinate systems differ significantly from geodetic coordinate systems, INCF DAI design borrows several key ideas from geospatial data infrastructure. As in geodesy, DAI recognizes a number of coordinate systems in different atlases, and does not mandate a single reference space. At the same time, WHS, being a publicly available open reference space, serves as a common and convenient “go-between” system much like latitude and longitude coordinates in a well-defined SRS (e.g., WGS84) are often used to transform coordinates between any two arbitrary systems. This allows us to use space rather than structural naming conventions to convey location. Structure names then become a type of information, which may be available at a location in the space of the brain, and may be different across atlases. For example, the same point location may be labeled as “Striatum dorsal region” in the Allen Mouse Brain Atlas, “Caudate putamen striatum” in the Paxinos atlas, or “Striatum” in WHS (**Figure 9B**), with names generally depending on image modality, delineation techniques, classification model, or adopted level of generality.

To create spatial infrastructure for brain atlases, we:

- developed a generic representation of a rodent brain coordinate space,
- compiled a registry of such coordinate systems,
- computed transformations between several existing reference spaces and implemented them as a set of standard services, and
- composed and implemented a workflow for deriving new coordinate systems and associated transformations between the new coordinate system and an existing one.

**Figure 3:**
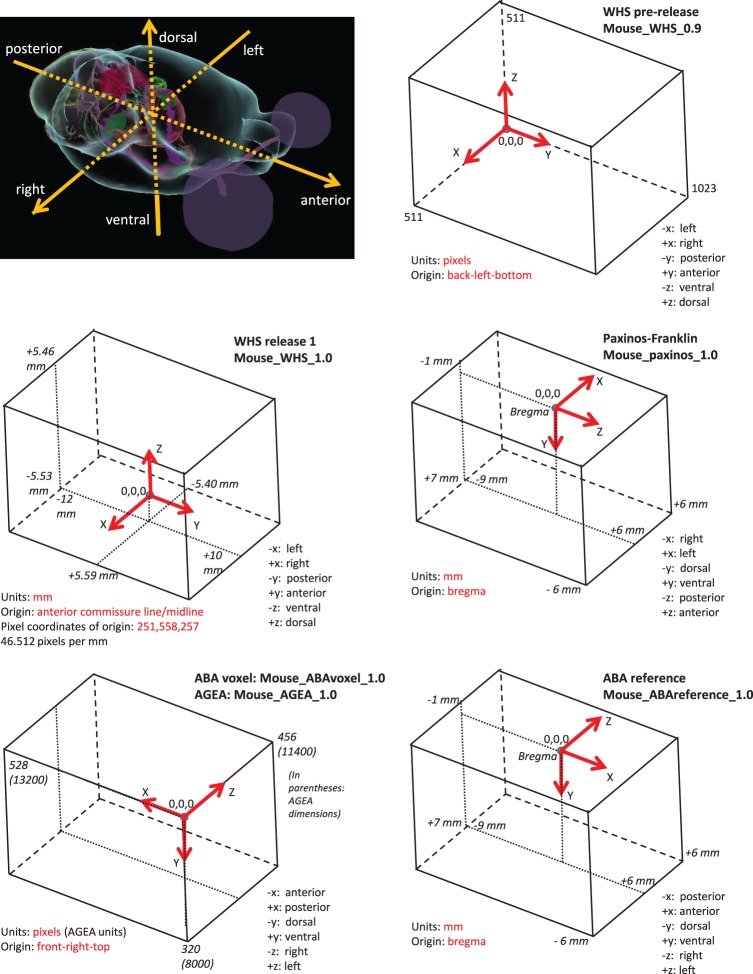
**Selected coordinate systems for mouse brain of several common atlas reference spaces**. All coordinate systems (boxes) are shown relative to the anatomical picture of the mouse brain shown in the upper left corner. Note the variability in direction and origin of the atlases. Much of the variability arose from practical reasons (e.g., stereotaxic surgery) or because of the data collection method used.

Table 1 lists several of the coordinate systems for rodent brain initially defined by the project and included in the SRS registry. These came from members of the atlasing community that were able to fairly quickly share their data within a spatial framework (e.g., User 3). Figure 3 illustrates some of them, along with origin and axis orientation shown on each diagram with respect to neuroscience orientations, as well as units and spatial extent on each coordinate axis. Note the wide variability in coordinate systems used in the various atlases.

**Table 1:** **Spatial reference system core characteristics for the mouse atlases currently registered in DAI**.

**Code**	**Name**	**SRS family**	**Version**	**Species**	**SRS description**
INCF:0001	Mouse_WHS_0.9	WHS	0.9	Mouse	WHS initial version, with origin in the back-left-bottom corner
INCF:0002	Mouse_WHS_1.0	WHS	1.0	Mouse	WHS with origin shifted to the intersection of midline and the center of anterior commissure
INCF:0100	Mouse_ABAvoxel_1.0	ABAvoxel	1.0	Mouse	SRS used in the Allen Mouse Brain Atlas 3D model (circa 2005)
INCF:0101	Mouse_ABAreference_1.0	ABAreference	1.0	Mouse	SRS in the Allen Mouse Brain Atlas reference atlas
INCF:0102	Mouse_AGEA_1.0	AGEA	1.0	Mouse	SRS used in the Allen Mouse Brain Atlas gene expression module, a derivative of ABAvoxel
INCF:0200	Mouse_Paxinos_1.0	Paxinos	1.0	Mouse	SRS in the Paxinos and Franklin (2001) stereotaxic atlas of the mouse brain
INCF:0300	Mouse_EMAP-T23_1.0	EMAP-T23	1.0	Mouse	A T23 model of EMAP developing mouse atlas

In the current DAI model, SRS descriptions are designed to provide sufficient information for neuroscientists to understand how the SRS is constructed with respect to neuroscience orientation and key anatomic features, and evaluate its applicability as an alignment target. Therefore, SRS descriptions include:

- coordinate system origin,
- coordinate axes and measurement units,
- pointer to the SRS's reference implementation,
- specification of the region of validity and valid extents along each of the coordinate axes,
- the author of the SRS, and
- how the SRS was derived from another coordinate system, if applicable.

The “Region of Validity” is a characteristic analogous to the “Area of Use” in the EPSG registry. In addition to the whole brain coordinate systems registered so far, DAI allows users to register additional SRS defined more precisely for smaller regions in the brain, using the workflow described later in the paper. For such SRS, the region of validity is defined by an anatomic structure or a group of structures, and valid spatial extents along the X, Y, and Z axes. The DAI ability to manage multiple coordinate systems, both for the whole brain and local to an anatomic structure, facilitates spatial integration of neuroimaging information across different modalities and resolution levels, as DAI users can select an appropriate reference space (e.g., with matching resolution, region of validity, and modality) to explore available data or to register their own data.

The coordinate system registry contains an additional mandatory table called “Orientation,” which provides interpretation of neuroscience coordinate axes or their derivatives used to define X, Y, and Z coordinates in the SRS table. These axes may be simple (e.g., describing straight dorsal-ventral, anterior-posterior, or left-right orientations), or complex. The latter could be used to describe orientations in the developing brain (where the posterior and anterior orientations may be described as curves rather than straight lines) or volumes/images that are tilted or otherwise transformed with respect to canonical anatomical terms of location. Note that such a description should be sufficient for neuroscientists to understand how the coordinate system was constructed, and roughly orient it with respect to other SRS, but in most cases will be insufficient for deriving coordinate transformations: the latter are computed and registered separately.

Additional tables in the SRS registry are optional and include: “Structure,” “Fiducial,” and “Slice.” “Structure” includes descriptions of anatomic structures delineated in 2D or 3D, along with references to structure vocabulary and a spatial object describing the structure, or a method for deriving the latter. “Fiducial”s are recognizable points or higher-dimensional features generally derived from anatomic structures or their relationships, which can be used to automatically relate one SRS to another, or recommend point pairs for fine alignment. Finally, “Slice” is used when the SRS is defined through a collection of 2D plates with segmented structures rather than by a 3D volume; it contains descriptions of individual slices, or plates, that together form the 3D atlas. A more complete description of tables in the SRS registry can be found at http://wiki.incf.org/mediawiki/index.php/SRS_Registry.

In INCF-DAI, information from this registry (encoded in WaxML) is currently available via several atlas service requests that are supported by all atlas hubs (*ListSRSs* and *DescribeSRS*). WaxML and the atlas services are described in subsequent sections of the paper.

In addition to the registry of SRSs, INCF-DAI also maintains a registry of coordinate transformations between known coordinate systems. While there is no requirement for a specific coordinate system to be implemented by all atlas sources, there is a requirement that any new user-supplied atlas data are registered to at least one known coordinate system. For practical reasons, within INCF-DAI it is recommended that at least forward and inverse transformations between all SRSs and WHS are supported, since, with WHS as an intermediary, coordinate transformation between any two SRSs that do not have direct mapping, would require two steps. While this is not a strict requirement within DAI, limiting the number of steps in a composite transformation reduces any mapping errors that might occur due to registration.

Different procedures, depending on the representation (collection of 2D slices, 3D model) and known relationships between reference spaces, have been used to derive forward and inverse transformations between pairs of registered coordinate systems. Registration methods include those implemented in ITK/ANTS (Avants et al., 2011) (http://www.picsl.upenn.edu/ANTS) for 3D volume registration, warping of individual 2D slices to matching slices in a 3D volume using thin plate spline calculations, and piecewise linear mapping functions for selected 3D atlas slices to a 2D plate. In the absence of good assessment techniques for transformation accuracy between two images (besides visual inspection of resultant alignment), inverse transformation consistency is computed for each translation function and returned to the user as part of coordinate transformation responses (*TransformPOI*). Using the spatial alignment workflow provided within DAI, or any other similar workflow, users are encouraged to develop new transformations or additional versions of existing transformations to improve registration and coordinate transformation accuracy for their region of interest, make them available via atlas services, and register them in the registry of transformations.

### Waxholm markup language

Existing atlases often present examples of different implementations of closely related functionality, or multiple ways of encoding similar types of data. For example, gene expression information might be labeled as “high,” “low,” or “none” within a neural structure or quantified as a number in a structure or region of space. An example is the information available from Allen Brain Atlas's AGEA (Anatomic Gene-Expression Atlas) via its *GeneFinder* requests, which return numeric normalized expression value at a location in space (see http://help.brain-map.org/download/attachments/2818169/InformaticsDataProcessing.pdf?version=1&modificationDate=1319667590884, p. 5–6). In contrast, the Embryonic Edinburgh Map Atlas project (EMAP) framework holds EMAGE data, where expression levels are returned with keywords for a selected region such as “strong,” “detected,” or “not detected” (Baldock et al., 2003; Christiansen et al., 2006). This is likely the more common way of representing this type of information, but even these designations may be assigned using various methods. At the same time, there have been several efforts to develop gene expression markup, including MAGE-ML (Spellman et al., 2002) (http://www.mged.org/Workgroups/MAGE/mage.html), and MINiML (Barrett et al., 2007) (http://www.ncbi.nlm.nih.gov/geo/info/MINiML.html). This illustrates some of the diversity of perspectives, research approaches and methods of neuroscientists. Conveying information about both the methods and results in a formal schema that is human and machine readable and also acceptable to different atlas publishers is highly desirable, but extremely difficult. As discussed above, our strategy to overcome this hurdle is to develop an information system that supports convergence to a consensus representation rather than mandates a single representation from the start. While allowing atlas hub providers a degree of freedom, this approach recommends standard structures and semantics appropriate for exchange of spatial information in the brain and also allows continual updating and improving of representations as methods and analyses evolve.

WaxML is the information model used to express key elements from atlas hubs. It offers formal semantics for atlas information, defining valid elements, their attributes and relationships. Specifically, it provides type definitions for basic atlas classes that describe SRSs, spatial transformations and key geometry types (Table 2). It also gives output schemas for brain location-based service requests, which include structures for anatomic features, gene expression, images and image collections, annotations, and other objects returned in response to POI-based requests. As mentioned above, we allow for differently structured responses to similar requests, due to specific implementations and approaches adopted by different atlases, as long as geometric representations remain consistent and interoperable.

**Table 2:** **Common WaxML schema components (see https://code.google.com/p/incf-dai/)**.

**Schema name**	**Description**
CoordinateTransformationCommon	Constructs related to coordinate transformation information, including transformation code, implementing atlas hub, input SRS, output SRS, transformation performance, order of transformations in a transformation chain
SrsCommon	Constructs related to spatial reference systems (SRS), as described in Section Common Spatial Framework
WaxML_Base	Basic constructs used across WaxML, specifying base input and response types, geometry types, and key enumerations

**Figure 4:**
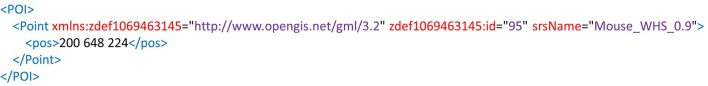
**Representation of point of interest (POI) using the GML Point construct**. Note that spatial reference system name is a mandatory attribute of Point.

**Figure 5:**
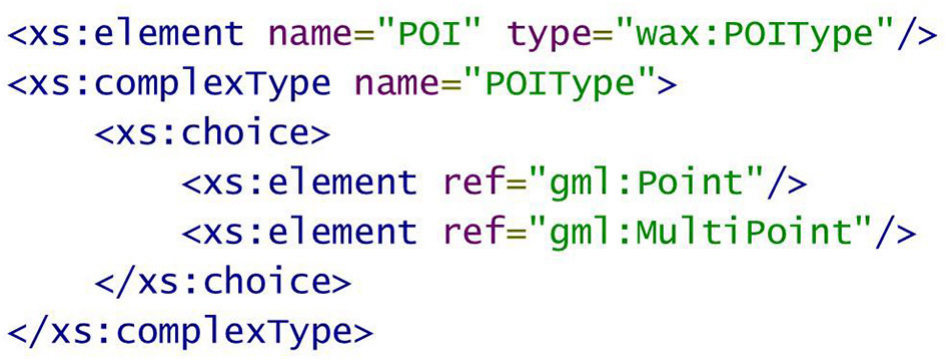
**Fragment of WaxML_Base.xsd schema referencing GML Point and MultiPoint constructs**.

WaxML borrows spatial object descriptions from the Open Geospatial Consortium (OGC) Geography Markup Language, GML (Portele, 2007), an international standard for spatial data encoding (ISO 19136). In particular, representation of spatial features and locations in the brain follows the GML simple features profile (Van den Brink et al., 2012). For example, a GML Point construct is used to encode points of interest (POI) (Figure 4), following POI definition in WaxML schema (in WaxML_Base.xsd), which references GML representation of points and multipoints—the latter construct is used when the request is to process an array of points rather than a single point of interest (Figure 5).

As an application schema of GML, the WaxML schema is compiled with GML 3.2.1, which is available at http://schemas.opengis.net/gml. Leveraging proven and well-documented standard geometric descriptions allows WaxML developers to reuse a range of common open source software libraries, and create software interoperable with multiple existing client and server codes, while focusing on classes that are specific to brain atlases.

### Atlas services

The atlas service interface specification is another key standard that forms the backbone of INCF-DAI. Atlas services are web functions that support querying and updating brain atlas resources offered by an atlas hub, returning information in WaxML-encoded documents.

The atlas services follow OGC Web Processing Service (WPS) interface standard (http://www.opengeospatial.org/standards/wps), which provides a framework for describing, invoking and chaining web requests, specifically oriented to spatial data processing functions. The key advantage of WPS for atlas services at this stage is that the services are self-describing (via the mandatory *GetCapabilities* and *DescribeProcess* requests), and the descriptions include information about the inputs and the output schema. The set of service requests may vary between atlas hubs, reflecting differences in implementation of atlas resources. Adherence to the WPS standard establishes initial structural consistency across different atlas services, and lets application developers reuse multiple standard service libraries (including WPS authoring libraries in Java and Python), client applications, and service metadata registries.

The general format of a WPS request is:

**http://<server-path>/<HostServiceController>?Service=WPS&version=1.0.0**
**  &Request=<WPS_Request>**
**  &Identifier=<identifier_name>**
**  &ResponseForm={format}**
**  &DataInputs={Encoded Inputs}**

where WPS_Request may be one of *GetCapabilities, DescribeProcess* or *Execute* statements; the <identifier_name> clause refers to the function (process) to be invoked, such as *Get2DImagesByPOI;* ResponseForm specifies the output format of the response; and DataInputs includes a list of input values.

The WPS standard, and standard libraries implementing WPS, offers a few additional capabilities useful for DAI, including the built-in ability to manage large volume processing on servers without returning processing results to the client application (via an optional &*storeExecuteResponse=true* clause), execute chains of functions, request status updates for long-running processes (via the optional &*status=true* clause), and return lineage information in service responses (via the optional &*lineage=true* clause).

A number of core and optional INCF-DAI atlas service requests have been defined, as described below (see http://wiki.incf.org/mediawiki/index.php/Atlas_Services for additional details).

#### Core atlas service requests

These atlas service requests include key operations enabling exchange of location information in DAI. They provide basic information about hub capabilities and supported functions as well as coordinate systems and transformations, and enable execution of transformations and transformation chains.

- Service capability descriptions: *GetCapabilities* and *DescribeProcess*. These requests, mandated by the WPS standard, provide a list of functions (processes) included in an atlas service, and their descriptions.
- Descriptions of SRSs hosted by an atlas service implemented at an atlas hub: *ListSRSs, DescribeSRS*. These requests return coordinate system origin, units, definitions of coordinate axes and other SRS metadata (see Common Spatial Framework) formatted as WaxML documents. The functions are implemented at all atlas hubs that publish data in a coordinate system unique to that hub.
- Spatial transformations: *ListTransformations, TransformPOI*. The first of these functions lists forward and inverse coordinate transformations implemented at a hub. Additional coordinate systems and transformations can be automatically added to the system as new images and volumes are registered using the registration workflow described in Section Data Publication: the Spatial Registration Workflow. The second function executes a specified transformation for given coordinates of a point of interest (POI) or an array of points, generating coordinates of the POI or a POI array in the target atlas space.
- A client application may request a coordinate transformation that involves several steps. For instance, translating coordinates between reference plates in the Paxinos mouse atlas in stereotaxic coordinates, and reference plates of the Allen Mouse Brain Atlas, requires a chain of transformations that involve WHS, AGEA, and Allen Mouse Brain Atlas voxel model as intermediary coordinate spaces. An optimal transformation path is generated by *GetTransformationChain* at the central atlas hub, as described in Section Implementation. This chain could be avoided if direct registrations existed between all of the reference atlases; however, this is not practical, so in many cases this direct mapping does not exist.
- Some atlas hubs may provide sparse content for certain types of data, hence atlas queries may return empty responses. For example, requesting annotations or 2D images available at a given POI may yield empty responses, especially in the early phases of DAI development. To optimize POI-based requests, general information about availability of different types of registered objects (images, annotations, gene expression data, etc.) in the vicinity of a given POI, across multiple atlas hubs, should be available. This information is returned on the *GetObjectsByPOI* request implemented at the central atlas hub, which returns a list of POI-based methods that would result in non-empty responses.

#### Optional atlas service requests

These atlas service requests are not mandatory but are likely to be implemented at one or several atlas hubs. Typically, these additional requests for individual hubs reflect information content provided by the atlas, and are implemented as WPS service wrappers over existing native functionality of the atlas resource.

These include such POI-based requests as *GetStructureNamesByPOI, Get2DImagesByPOI; GetCorrelationMapByPOI; GetGenesByPOI, GetAnnotationsByPOI*, which accept a point of interest in any known SRS and return a respective WaxML document from a given atlas service. For example, the *GetStructureNamesByPOI* method supports structure lookup for a canonical set of segmentations defined for an atlas, returning WaxML descriptions of structures found in the vicinity of a POI, along with geometric properties of each structure if available. While at this stage DAI is primarily concerned with coordinate information exchange and spatial requests (e.g., POI-based requests), atlas hubs may also include queries that don't involve brain location, e.g., queries by structure name, gene name, or similar.

As discussed earlier, the ability to have different sets of functions published by different hubs is a design requirement of DAI, as the initial goal is to standardize treatment of coordinate systems and location information, and create a framework in which the community can converge, over time, toward a common set of POI-based functions, related semantic functions, and the structure of requests and returned schemas.

## Implementation

As discussed earlier, a working prototype of INCF-DAI is implemented as a network of atlas hubs hosting atlas web services, the central metadata registry, which maintains a catalog of atlas resources, and a number of client applications that consume atlas service requests and use the results to integrate information from atlas hubs for analysis and visualization (Figure 2). These components are described below.

### Atlas hubs

The atlas services have been implemented for five hubs: Allen Brain Atlas mouse hub, UCSD Cell-Centered Database hub, Edinburgh Mouse Atlas Project hub, a WHS mouse hub, and a central INCF atlas hub. In addition, rudimentary services with minimum set of functions have been setup for the two WHS rat hubs discussed earlier, though POI-based requests are not yet available for them. Any group that also wants to share their spatially-linked data in this manner may also consider setting up an atlas hub (User 3). As outlined in Section Core Atlas Service Requests, the hubs present service capability descriptions, SRSs unique to the hub, and coordinate transformations between these SRS and one or more globally-known coordinate system, such as WHS. The criterion is that for each hub publishing atlas data in a unique SRS, there should be at least one set of forward and inverse transformations that can be ultimately (i.e., via a sequence of transformations) connected with WHS, which in turn is maintained at the WHS hub. For example, the Allen Brain Atlas hub publishes three coordinate systems; the Allen Mouse Brain Atlas reference plates (ABAreference), Allen Mouse Brain Atlas 3D volume (ABAvoxel), and AGEA, in addition to several pairs (forward and inverse) of coordinate transformations: between ABAreference and ABAvoxel, between ABAvoxel and AGEA, and between ABAvoxel and WHS.

Besides these core functions, atlas hubs publish different sets of service methods, typically implemented as WPS wrappers over native atlas functions offered by their databases. For example, the ABA hub includes such functions as *Get2DImagesByPOI; GetCorrelationMapByPOI; GetGenesByPOI*, which wrap native ABA or AGEA functions (e.g., AGEA's GeneFinder interface takes coordinates of a seed point in AGEA coordinates as input).

In addition to hubs that publish specific atlas resources and/or coordinate systems and transformations, there is a special “central atlasing hub,” which serves as a query mediator across other hubs and manages coordinate translations that involve more than one hub. It hosts a standard set of WPS-based atlas functions, which accept POI-based requests and translate them into respective web service requests against all registered hubs, then unions the responses before returning them to the user application. For example, a user may request a list of all 2D images available for a particular part of the brain from all atlas sources that support the *Get2DImagesByPOI* (illustrated in **Figure 9**). Information about all hubs that support this request is available because the atlas web service has been registered in the central service registry (see The INCF Central Metadata Registry and Discovery Portal for Atlas Resources), and lists of supported functions from each hub have been harvested into the central catalog. With this information available to the mediating hub, it rewrites the initial *Get2DImagesByPOI* query into respective requests that are valid for each atlas source.

**Figure 6:**
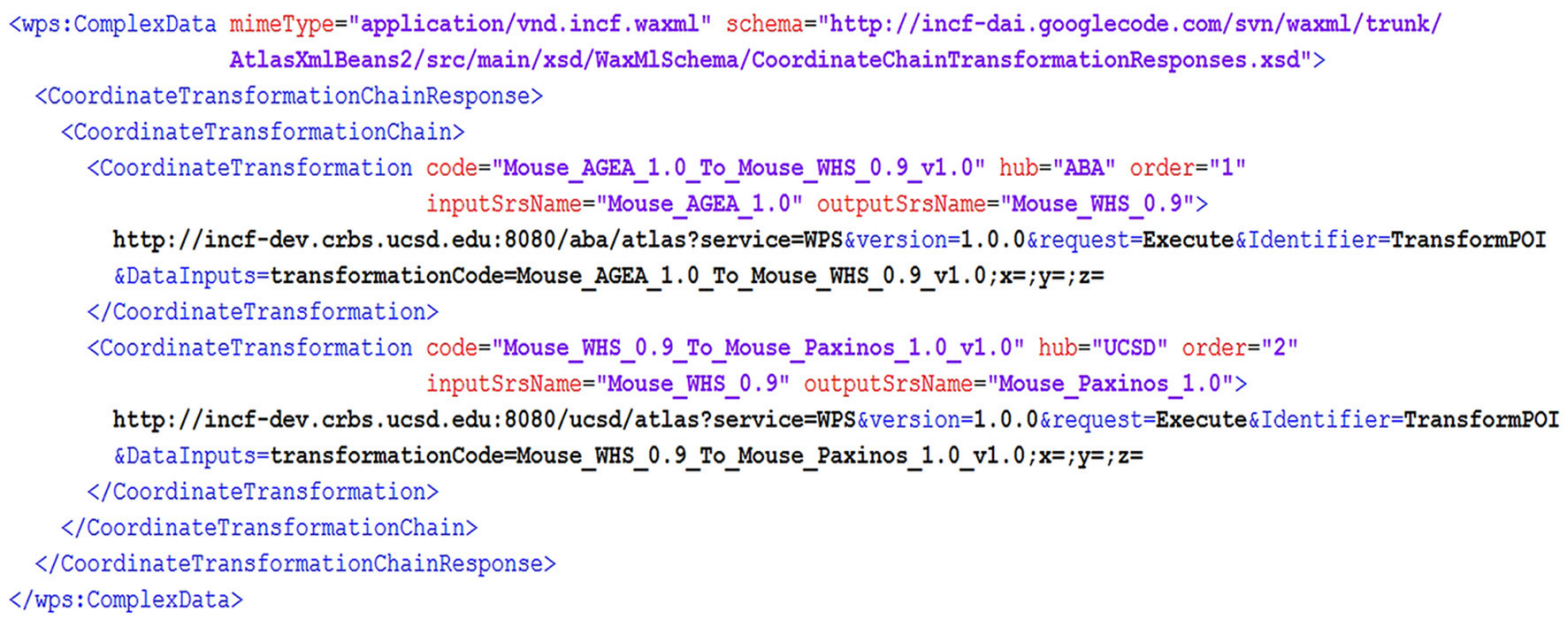
**A fragment of *GetTransformationChain* response**. The response describes transformations from the Allen Mouse Brain AGEA (Mouse_AGEA_1.0) to the coordinate space developed in the (Paxinos and Franklin, 2001) mouse brain atlas (Mouse_Paxinos_1.0). It includes two *TransformPOI* request templates (with X, Y, Z coordinates left blank) served by two different atlas hubs: the ABA hub and the UCSD hub. The two *TransformPOI* service requests need to be made in sequence to execute the transformation chain. Note that the Mouse_WHS_0.9 coordinate space serves as the intermediate space for the two transformations: from AGEA to WHS 0.9 and then from WHS 0.9 to the target reference space of (Paxinos and Franklin, 2001).

An additional useful feature of DAI is that information for POI in the brain can be requested in any known coordinate system, since SRSName is a mandatory part of a POI definition. Coordinate translation to SRS understood by each hub are performed automatically, with the help of the *GetTransformationChain* request implemented at the mediator hub. This request uses information about all registered coordinate systems (which is harvested into the central database from all atlas services via *ListSRSs* calls) to construct an optimal sequence of coordinate translations from the POI included in user request, to target SRSs that a hub can process. The sequence of transformations is then executed as a series of TransformPOI calls. This processing is done behind the scenes, effectively allowing users and applications to issue service requests against any POI-based service in any known coordinate system. For example, a service request may use a POI in the coordinates of the Allen Mouse Brain AGEA, and expect it to be translated into the coordinate space of the (Paxinos and Franklin, 2001) mouse brain atlas, for querying atlas hubs that support the latter coordinate system. The respective *GetTransformationChain* request will generate a series of coordinate transformations such as the one shown in Figure 6, which involve a sequence of TransformPOI requests at the ABA and UCSD atlas hubs.

In the DAI prototype project, we used Deegree WPS libraries (http://www.deegree.org/) to develop and configure atlas services. This open source software implements OGC WPS 1.0.0, and configures standard WPS *GetCapabilities* and *DescribeProcess* requests based on a list of process providers, which represent containers for processes (functions) written in Java. The initial processes to publish through this mechanism include *ListSRSs* and *DescribeSRS* functions. Next, the hub author generates forward and inverse coordinate transformations that connect each of the new SRSs with WHS or another previously registered coordinate system, and makes this information available via *ListTransformations* and *TransformPOI* functions. After that, additional POI-based requests are implemented as appropriate for the types of resources to be published through the hub, using the same Java process containers. Other WPS development libraries can be used as well, such as PyWPS (in Python, http://pywps.wald.intevation.org/) or ZooWPS (multiple languages, including C/C++, Fortran, Java, Python, PHP, Perl, JavaScript: http://www.zoo-project.org/).

### The INCF central metadata registry and discovery portal for atlas resources

INCF Atlas Central, hosting INCF-DAI portal and catalog, and a set of central registries (metadata, list of reference spaces and transformations) is the primary metadata registration, discovery, and integration platform. It is configured to periodically harvest information from individual atlas hubs via *GetCapabilities, DescribeProcess, ListSRSs*, and *ListTransformations* requests.

Atlas service metadata, as well as metadata for other types of registered resources (atlas-related image services, web-accessible folders with file collections, individual downloadable files, web sites, offline data, other standard catalog services, etc.), is organized in a central catalog, which is compliant with an international standard for spatially-enabled catalogs called *OGC Catalog Services for the Web* (CSW) (http://www.opengeospatial.org/standards/cat). This standard defines the request and response protocol for searching, adding, updating, and deleting catalog records. This CSW catalog is the core component of the INCF-DAI portal. The portal is implemented using open source Geoportal Server (http://sourceforge.net/projects/geoportal/) software, which is pre-configured to recognize standard service descriptions such as WPS, supports regular harvesting and updating registered resources of known types, and lets users browse and query atlas resource online.

**Figure 7:**
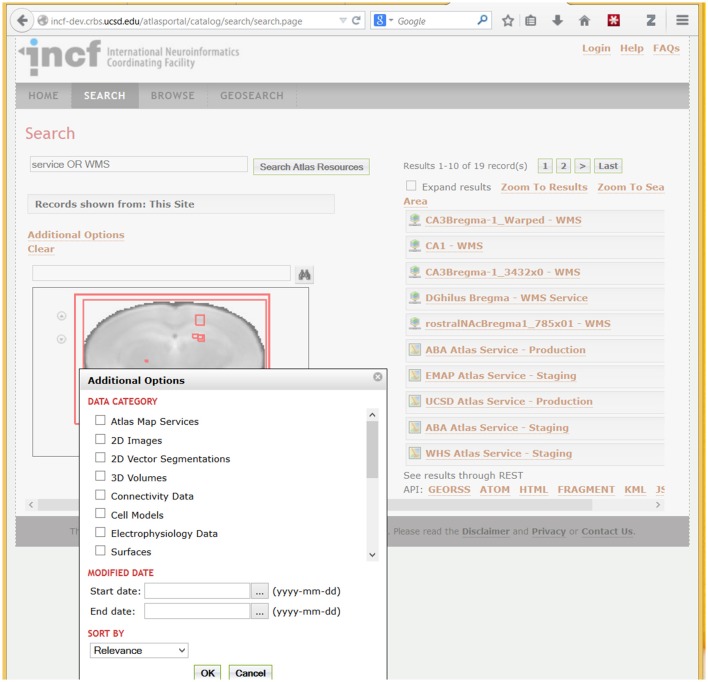
**A fragment of the DAI portal interface showing search results and types of searchable data**. The example search for “Service OR WMS” (in Search Atlas Resources entry) returns metadata records that contain these terms. WMS refers to the OpenGIS Web Map Service standard (http://www.opengeospatial.org/standards/wms), which is used by the UCSD Cell Centered DataBase (UCSD Hub) to provide online access to large spatially-registered 2D images; thus all images stored using this method are returned in this search. Spatial extents of the found resources, in brain coordinates, are shown as red rectangles over a coronal slice. Users can optionally search for specific atlas data types (under “Data Category”) illustrated in the pop-up box in the lower left corner. In addition to search, the portal supports metadata browsing (under the Browse tab) and search of resources based on geographic location of the lab that published a resource (under the GeoSearch tab).

We have customized the portal to support atlas-specific data types such as 2D images, segmentations, 3D volumes, connectivity data, and segmentations (Figure 7) and integrated it with several atlas client applications including WIB and Scalable Brain Atlas visualization clients. Because of the adoption of the CSW standard, the portal can be easily federated with other CSW-compliant portals, so that resources registered with one of the portals can be queried through another one.

### Client applications accessing atlas web services

**Figure 8:**
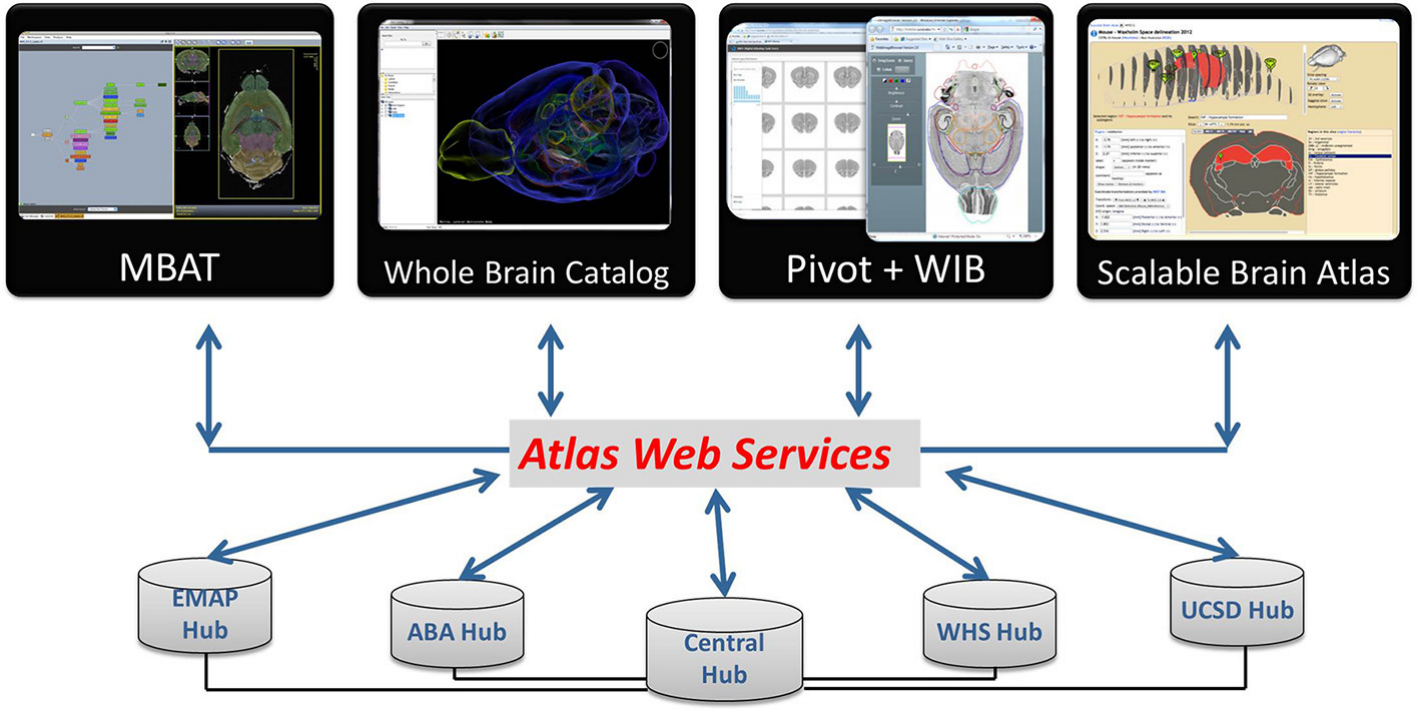
**DAI resources can be accessed via atlas web services from a number of atlas applications**. Users can find what is available from INCF Central, and query atlas hubs via the Central Hub or directly through their web services. Online applications accessing atlas resources (the Whole Brain Catalog, PivotViewer, WIB, Scalable Brain Atlas) are available from the DAI portal.

**Figure 9:**
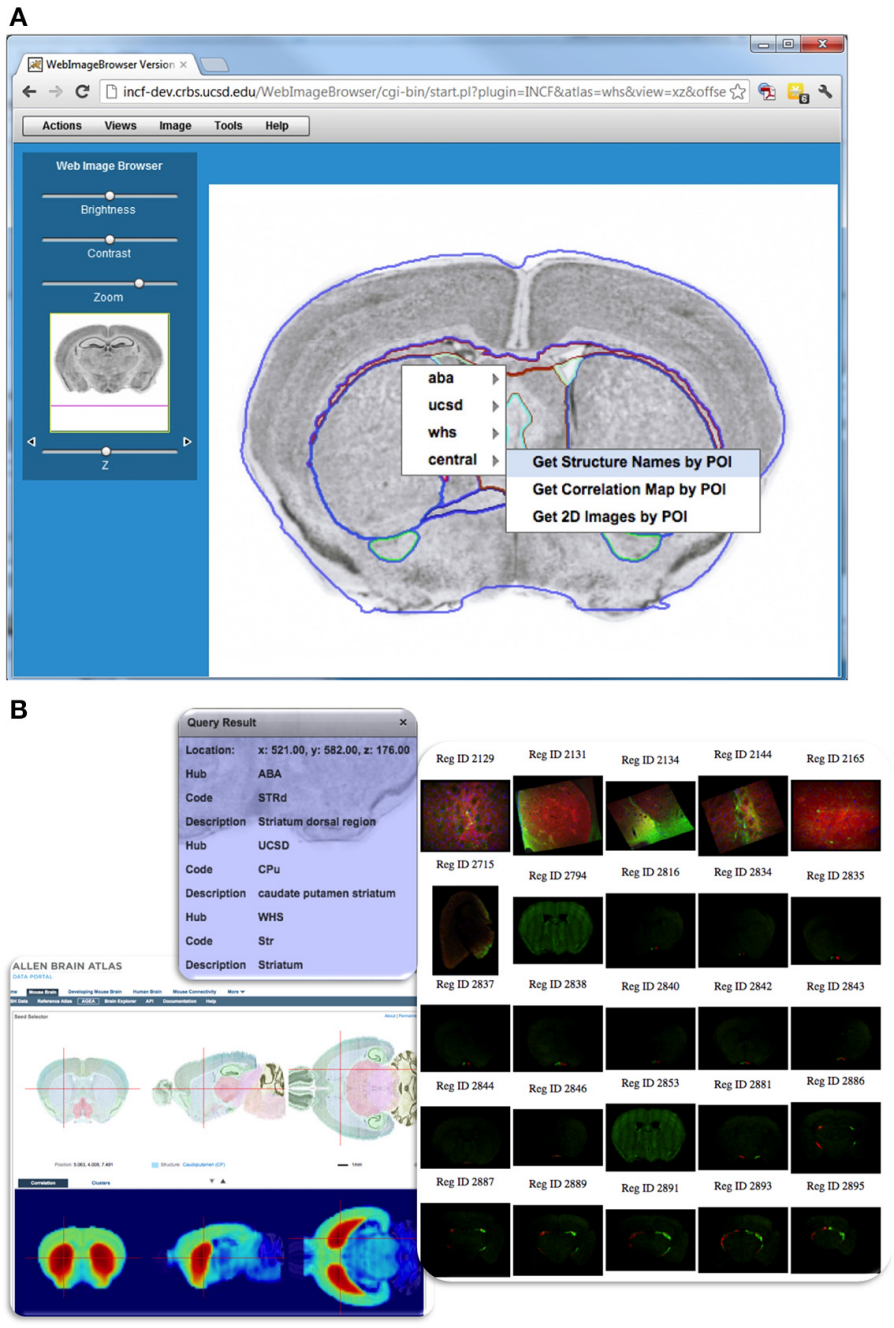
**Querying DAI resources using POI-based requests in WIB**. **(A)** Web Image Browser (WIB), illustrates how one can query the different atlases from a user-selected POI. As the user browses to a location of interest in the dataset and selects a POI for query, a menu appears showing registered atlas services and functions offered from each hub. Items in the menu invoke POI-based service functions, which return the requested information to the user. The outlines of structures from a reference atlas aid the user during navigation. **(B)** Example query results showing structure names from several atlases, gene correlation map served by Allen Brain Atlas, and spatially registered images near the POI served by CCDB.

Besides the atlas portal, resources registered in DAI can be accessed from a number of web applications (several shown in Figure 8). These applications make use of atlas service methods including coordinate translations and POI based requests. For example, WIB (Orloff et al., 2013) allows users to browse multiple atlas sections in three dimensions, and displays segmented anatomic features over high-resolution brain images (Figure 9). Users can zoom in to a POI and use it to query available atlas services and retrieve resources available from individual atlas hubs, or through the “central” atlas service, which spawns requests to all registered hubs and unions responses in a single output. The DAI coordinate translation services (*TransformPOI*) have also been used in the Scalable Brain Atlas (Bakker et al., 2010) (http://scalablebrainatlas.incf.org/), the Mouse BIRN Atlasing Toolkit (MBAT) (Ruffins et al., 2010) and the Whole Brain Catalog (Larson et al., 2010) (www.wholebraincatalog.org). In addition, a Python API accessing atlas web services has been developed (http://software.incf.org/software/incfdai?searchterm=python+DAI).

With these applications, users can compare anatomic feature descriptions, gene expression and other types of data available in different atlases and at different locations of interest. The Python wrapper also makes it easy for researchers to develop their own applications that take advantage of atlas services and the DAI framework.

## Data publication: the spatial registration workflow

The key DAI challenge is making the system extensible, to let users easily register and align their own data with existing atlases, add coordinate systems and transformations, and contribute additional data to an atlas hub. This is usually done to expand analysis options and/or to allow direct comparison to other spatially-linked resources (User 2). Thus, the system would not be complete without a prototype registration workflow for aligning user-supplied 2D images and image collections to INCF-DAI reference spaces. While image alignment tools and pipelines have been developed (e.g., ITK/ANTS, LONI Pipeline, Amira, Slicer, NeuroMaps, MBAT, etc.), they often can be difficult to install, only accept 3D volumes, or the registration transformation is not stored along with the original datasets in an easily accessible and reusable manner.

Our goal was to develop a lightweight and intuitive online registration system for individual 2D images that uses a slice of a canonical atlas as the target. The system would be able to process images that are poorly aligned or have other artifacts preventing a straightforward 3D reconstruction; and would generate DAI SRS descriptions and transformations that are stored in association with the dataset, as the workflow outcome. This last step is essential to being able to reuse this information for analytic or query purposes.

**Figure 10:**
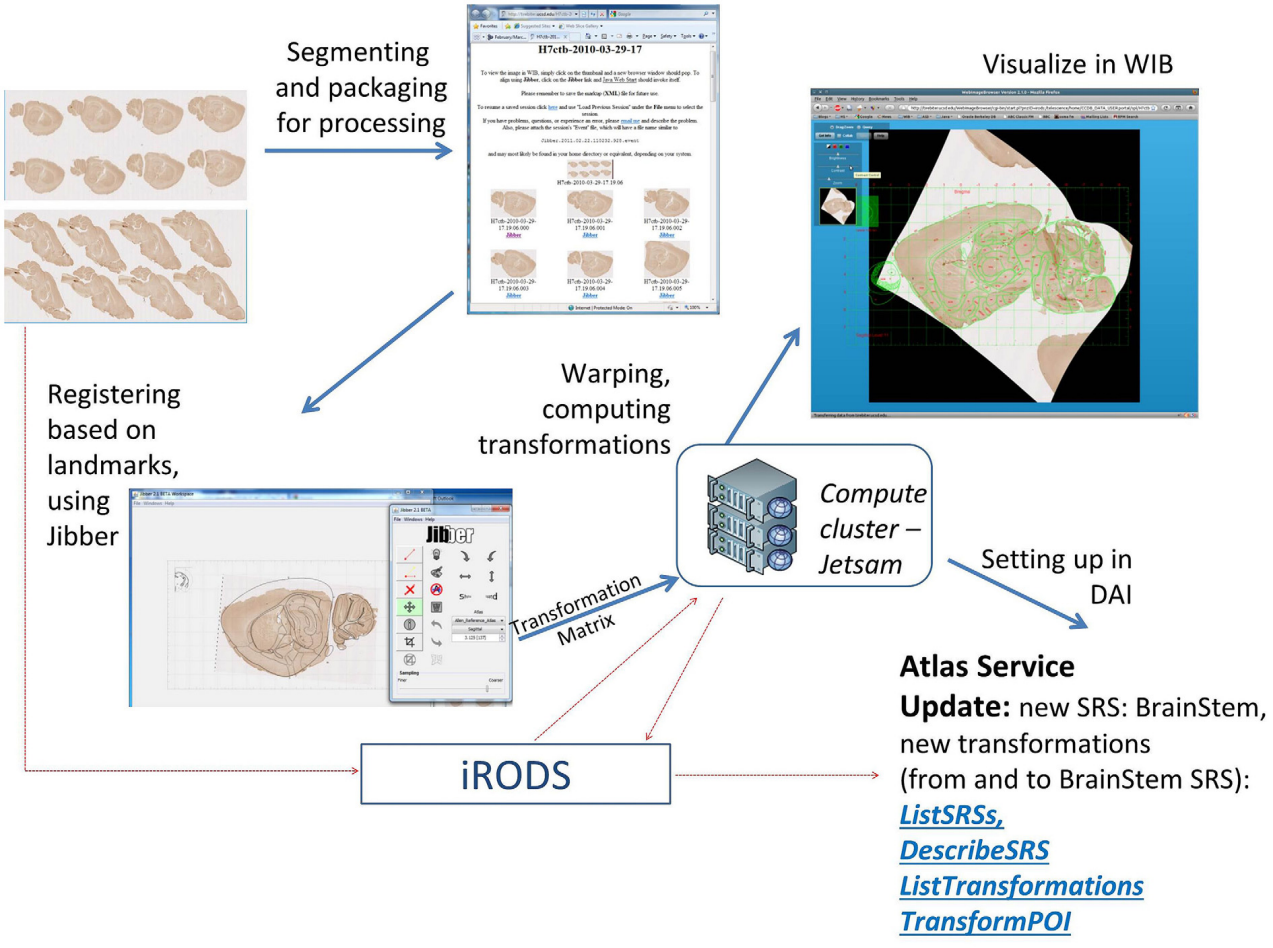
**Main steps of the atlas registration workflow for collections of 2D images**. The example images are from a study of innervation and genetic similarity in brainstem (Matthews, 2012). The images are segmented, packaged together and uploaded to INCF DataSpace. Subsequent steps include generation of an image gallery page, aligning individual images in the gallery with target reference plates (using Jibber), generating thin plain spline transformations, generating warped images (using Jetsam), generating and updating a new SRS description (called BrainStem) and forward and inverse transformations between the new SRS and the target reference atlas (in this case, the ABA reference atlas). Once the user has registered their data, they can identify areas of interest in their datasets and apply information from other Atlas Hubs to their data (e.g., what structure is found at this location in space in the Allen Brain Atlas). More analytic capabilities are also possible, but these are not currently offered by the INCF Digital Atlasing Program.

This workflow can be accessed from the atlas portal, but requires an INCF account. The main workflow steps are shown in Figure 10. In the first step, a collection of segmented images is uploaded into INCF DataSpace (http://www.incf.org/resources/data-space) via the INCF Atlas portal. The INCF DataSpace represents a common virtual storage space, where data from different INCF-affiliated labs are organized logically, abstracting specific storage resources used by each lab. It is implemented using iRODS (http://irods.org), which supports rule-based management of distributed files and file collections. In the context of INCF-DAI image registration workflow, iRODS rules are used to invoke initial processing of the uploaded images or image collections: generation of image pyramids, sub-sampled versions of the images, and image thumbnails. In addition, a *manifest* file is created, holding basic provenance information about the uploaded file collection and the processing steps.

Once the image files are packaged for processing, the content of the manifest file, and associated image thumbnails, are presented to the user in an image gallery page. From this page, users can visualize images in WIB or invoke the alignment interface. The latter component loads a sub-sampled version of the selected image into an alignment tool called *Jibber*. Jibber lets the user select a matching reference plate from a canonical atlas (in the current version, Allen Brain Atlas mouse reference plates or WHS sections), then adjust the image to match the target atlas plate as closely as possible. The affine transformation steps are followed by thin plate spline transformation based on user-defined links that connect correspondence point pairs or *tie-points* on the image and the target atlas plate. The generated transformation coefficients are passed to an engine called *Jetsam*, which generates a warped image and stores it in iRODS. The warping engine has been implemented on a computer cluster, to ensure fast warping of very large images. Based on these computations, a coordinate system description is generated, along with forward and inverse transformations between the user-submitted images and the canonical atlas used as the registration target.

The SRS description and the transformations are updated as additional images from the image gallery are registered. This allows users to query other DAI information using spatial locations on their own images to retrieve structure names, discover available registered images, or explore gene expression and other data associated with user-defined POI, using an online tool such as WIB (Figure 9).

## Using DAI

In addition to DAI technical components we have also developed tools and documentation to aid both neuroscientists and software developers interested in using or extending the system. Here we describe how these different users can find resources to access and contribute to the DAI.

The three types of neuroscientist users whose needs are addressed by DAI, are discussed in the introduction. User 1 wants to find and examine information about their area of interest, User 2 wants to compare their data to canonical atlases, and User 3 wants to contribute large datasets to a known spatial framework.

A simple query tool has been extended to fill the needs of User 1, WIB (see Section Client Applications Accessing Atlas Web Services); it can be found on the atlasing portal. The spatial registration workflow (Section Data Publication: the Spatial Registration Workflow) was created specifically to fit the needs of User 2. Finally, User 3 would need to first create an atlas hub, by setting up hub software, initially with a small set of mandatory atlas service functions, then defining additional spatial query functions appropriate for their data, and developing spatial transformations between hub's data and any other known SRS. Documentation on how to create at atlas hub can be found at http://code.google.com/p/incf-dai/wiki/HowToCreateAHub. The documentation points to general code libraries and hubs implemented within the project, which can be leveraged by software developers in creating new atlas hubs. The software, including WaxML schema, libraries, and coding examples is available at http://code.google.com/p/incf-dai, and can be used by developers wishing to build on any part of DAI. If resources allow in the future, we would create additional tools to more easily implement an atlas hub, at least for certain data types.

## Conclusions and future work

Today's neuroscientist is quite familiar with using interactive online maps to access diverse information from different sources. Tools like Google Maps are appealing because they serve as gateways to enormous amounts of spatially-registered information. This type of functionality, if available in the realm of neuroscience, would appeal to researchers, as everything is tied to “where in the brain” and relating different data by brain location would greatly facilitate our ability to do rigorous, and unique quantitative analyses (Carson et al., 2005; Kovacević et al., 2005; Christiansen et al., 2006; Leergaard and Bjaalie, 2007; Lein et al., 2007; Ma et al., 2008; Aggarwal et al., 2009; Ng et al., 2009; Chuang et al., 2011). Atlas projects of the Allen Brain Institute are a great example of what is possible when this kind of information is put within the context of spatial maps. Ideally, all neuroscience data would be presented within an accessible spatial framework such as this in order to facilitate our ability to find, analyze, and integrate diverse information. However, given multiple reference atlases developed with different functionality, data types, and spatial and semantic conventions, opportunities for researchers to easily access and integrate data from many of them, remain limited. Even more difficult, is the ability for most researchers to place their own data into a compatible spatial framework for comparison and analysis. This is becoming an acute problem with new techniques for 3D brain imaging such as microCT and methodologies for whole-brain fluorescent imaging (Susaki et al., 2014).

The purpose of this project is to fill the digital atlasing needs of neuroscientists who lack the resources to explore the rapidly growing collections of multidimensional atlas data based on brain location, compare their data with canonical atlases, or publish their data and make it accessible to others via spatial queries. Creating a data-rich and uniform spatial integration framework for atlas sources is challenging because of diversity across reference atlases, data types, and technologies, in addition to the lack of native spatial query functionality of atlas publishers. Thus, our solution has been to create a flexible and extensible framework that accepts existing resources, offers them formal descriptions, in addition to translations and spatial data exchange mechanisms between them.

The INCF-DAI framework addressed these atlas data integration challenges by developing information models for spatial references systems (SRS) in mouse brain; creating web-accessible registries of SRS and coordinate transformations between them, proposing a standard markup language for encoding SRS, and transformations. It offers the ability to query based on spatial location anatomic features and other common atlas constructs (returned via WaxML) through a system of atlas web services that communicate location information between atlas sources and clients. These components became the backbone of the prototype SOA for brain atlas data, which has been implemented via a collection of atlas hubs hosting web services, service metadata catalogs, central discovery portal, and a collection of atlas clients that use the services to perform coordinate transformations or retrieve information for a given POI. Since a broader consensus about community spatial integration frameworks for the brain is yet to emerge, a key requirement for the infrastructure prototype has been flexibility and extensibility of the specifications and their ability to incorporate different implementations of related functions.

This work demonstrated the power of leveraging spatial information integration resources that have been developed and standardized in other disciplines with longer history of managing and exchanging spatial location information. Reusing international standards for the description of spatial features such as GML, and spatial processing functions such as WPS, allowed us to streamline architecture development and create a more robust and maintainable system leveraging open source standards-compliant software. In addition, this helped us better understand the specifics of spatial representation and spatial information processing for brain data as compared to spatial descriptions used at the earth scales.

There are a number of challenges and limitations of the infrastructure prototype that should be addressed in future work. Ideally, we would be able to extend WHS and DAI approaches to other developmental phases and species, and fully explore the potential of spatial data integration. Relating information across phases and species would help address key research issues that underlay the use of all animal models of human neurological disorders. In addition, we would also like to create additional tools, resources, and documentation that reduces the effort needed for researchers to add their data to this framework, or to take advantage of it for their own analysis purposes.

More technical desired additions to the DAI include:

- Formal modeling of coordinate transformations that can accommodate different types of atlas references spaces.
- Consistent assessment of performance of coordinate transformations between atlas spaces, in particular evaluating quality of transformations and chains of transformations;
- Incorporating multiple ways of representing location in the brain (by coordinates, by anatomic feature name, by a collection of location rules, i.e., statements that include anatomic features and spatial relationships), and making such representations interoperable. This would be extremely useful for extending DAI to different developmental phases and species, where relating information by coordinates would be unreliable.
- Extending POI-based data exchanges to exchanging information for regions-of-interest, trajectories (along certain paths), transects, etc.
- Building community consensus about common data representation and functionality associated with atlases and further standardizing atlas services.

The latter typically requires significant time, effort and a formal and transparent process involving both neuroscientists and IT experts, which includes several phases: from identifying areas for standardization, to community review of proposed standards, pilot implementations and interoperability experiments, and to adoption and standards management. We believe that addressing atlas data integration challenges in a consistent manner, moving toward best practices and, eventually, community standards for atlas data representation and exchange, allows neuroscientists to more easily share data in a common spatial framework. This in turn, greatly increases accessible data and has the potential to facilitate data analysis, comparison, cross-validation, and integration across disciplines, developmental stages, and species. The work described in this paper offers first steps toward tackling many of the hurdles to sharing spatially-tied data as well as a framework that can be shaped and expanded by the research community.

### Conflict of interest statement

The authors declare that the research was conducted in the absence of any commercial or financial relationships that could be construed as a potential conflict of interest.

## Footnotes

^1^http://wiki.incf.org/mediawiki/index.php/Use_Case

^2^Named after Waxholm, a town in Sweden where the first meeting of the INCF atlasing task force was held in 2007.

## Abbreviations

ABA: Allen Brain Atlas
AGEA: Anatomic Gene Expression Atlas
API: Application Programming Interface
CSW: Catalog Services for the Web
DAI: Digital Atlasing Infrastructure
EMAGE: Edinburgh Mouse Atlas Gene Expression database
GML: Geography Markup Language
INCF: International Neuroinformatics Coordinating Facility
MBAT: Mouse BIRN Atlasing Toolkit
OGC: Open Geospatial Consortium
POI: Point of Interest
SOA: Service-Oriented Architecture
SRS: Spatial Reference System
WHS: Waxholm Space
WaxML: Waxholm Markup Language
WIB: Web Image Browser
WPS: Web Processing Service.

## References

[B1] Aggarwal M. Zhang J. Miller M. I. Sidman R. L. Mori S. (2009). Magnetic resonance imaging and micro-computed tomography combined atlas of developing and adult mouse brains for stereotaxic surgery. Neuroscience 162, 1339–1350 10.1016/j.neuroscience.2009.05.070 19490934 PMC2723180

[B2] Avants B. B. Tustison N. J. Song G. Cook P. A. Klein A. Gee J. C. (2011). A reproducible evaluation of ANTs similarity metric performance in brain image registration. Neuroimage 54, 2033–2044 10.1016/j.neuroimage.2010.09.025 20851191 PMC3065962

[B3] Bakker R. Larson S. D. Strobelt S. Hess A. Wojcik D. Majka P. (2010). Scalable brain atlas: from stereotaxic coordinate to delineated brain region. Front. Neurosci. Conference Abstract: Neuroinformatics 2010. 10.3389/conf.fnins.2010.13.00028

[B4] Baldock R. A. Bard J. B. L. Burger A. Burton N. Christiansen J. Feng G. (2003). EMAP and EMAGE A framework for understanding spatially organized data. Neuroinformatics 1, 309–325 10.1385/NI:1:4:309 15043218

[B5] Barrett T. Troup D. B. Wilhite S. E. Ledoux P. Rudnev D. Evangelista C. (2007). NCBI GEO: mining tens of millions of expression profiles–database and tools update. Nucleic Acids Res. 35, D760–D765 10.1093/nar/gkl887 17099226 PMC1669752

[B6] Bjaalie J. G. (2002). Localization in the brain: new solutions emerging. Neuroscience 3, 322–325 10.1038/nrn790 11967564

[B7] Boline J. Lee E. F. Toga A. W. (2008). Digital atlases as a framework for data sharing. Front. Neurosci. 2, 100–106 10.3389/neuro.01.012.2008 18982112 PMC2570073

[B8] Carson J. P. Ju T. Lu H. C. Thaller C. Xu M. Pallas S. L. (2005). A Digital atlas to characterize the mouse brain transcriptome. PLoS Comput. Biol. 1:e41 10.1371/journal.pcbi.0010041 16184189 PMC1215388

[B9] Christiansen J. H. Yang Y. Venkataraman S. Richardson L. Stevenson P. Burton N. (2006). EMAGE: a spatial database of gene expression patterns during mouse embryo development. Nucleic Acids Res. 34, D637–D641 10.1093/nar/gkj006 16381949 PMC1347369

[B10] Chuang N. Mori S. Yamamoto A. Jiang H. Ye X. Xu X. (2011). An MRI-based atlas and database of the developing mouse brain. Neuroimage 54, 80–89 10.1016/j.neuroimage.2010.07.043 20656042 PMC2962762

[B45] David P. A. Greenstein S. M. (1990). The economics of compatibility standards: an introduction to recent research. Econ. Innov. New Techn. 1, 3–42 10.1080/10438599000000002

[B11] Erl T. (2005). Service-Oriented Architecture. Vol. 8 New York, NY: Prentice Hall

[B12] Hawrylycz M. J. Baldock R. A. Burger A. Hashikawa T. Johnson G. A. Martone M. (2011). Digital atlasing and standardization in the mouse brain. PLoS Comput. Biol. 7, 2–7 10.1371/annotation/22c5808a-56cf-46e5-ba1b-456e838a5428 21304938 PMC3033370

[B13] Hawrylycz M. J. Boline J. Burger A. Hashikawa T. Johnson G. A. Martone M. (2009). The INCF digital atlasing program: report on digital atlasing standards in the rodent brain. Nat. Preced. 10.1038/npre.2009.4000.1

[B14] Hof P. R. Young W. G. Bloom F. E. Belichenko P. V. Cello M. R. (2000). Comparative Cytoarchitectonic Atlas of the C57BL/6 and 129/Sv Mouse Brains. Amsterdam: Elsevier

[B15] Johnson G. A. Badea A. Brandenburg J. Cofer G. Fubara B. Liu S. (2010). Waxholm space: an image-based reference for coordinating mouse brain research. Neuroimage 53, 365–372 10.1016/j.neuroimage.2010.06.067 20600960 PMC2930145

[B16] Johnson G. A. Calabrese E. Badea A. Paxinos G. Watson C. (2012). A multidimensional magnetic resonance histology atlas of the wistar rat brain. Neuroimage 62, 1848–1856 10.1016/j.neuroimage.2012.05.041 22634863 PMC3408821

[B17] Josuttis N. (2007). SOA in Practice: The Art of Distributed System Design. O'Reilly Media, Inc.

[B18] Kovacević N. Henderson J. T. Chan E. Lifshitz N. Bishop J. Evans A. C. (2005). A three-dimensional MRI atlas of the mouse brain with estimates of the average and variability. Cereb. Cortex 15, 639–645 10.1093/cercor/bhh165 15342433

[B19] Larson S. D. Aprea C. Martinez J. Little D. Astakhov V. Kim H. S. (2010). An open Google Earth for neuroinformatics: the whole brain catalog. Front. Neurosci. Conference Abstract: Neuroinformatics 2010. 10.3389/conf.fnins.2010.13.00137

[B20] Lee D. Ruffins S. Ng Q. Sane N. Anderson S. Toga A. W. (2010). MBAT: a scalable informatics system for unifying digital atlasing workflows. BMC Bioinformatics 11:608 10.1186/1471-2105-11-608 21176225 PMC3023809

[B21] Leergaard T. B. Bjaalie J. G. (2007). Topography of the complete corticopontine projection: from experiments to principal Maps. Front. Neurosci. 1, 211–223 10.3389/neuro.01.1.1.016.2007 18982130 PMC2518056

[B22] Lein E. S. Hawrylycz M. J. Ao N. Ayres M. Bensinger A. Bernard A. (2007). Genome-wide atlas of gene expression in the adult mouse brain. Nature 445, 168–176 10.1038/nature05453 17151600

[B23] Ma Y. Smith D. Hof P. R. Foerster B. Hamilton S. Blackband S. J. (2008). *In Vivo* 3D digital atlas database of the adult C57BL/6J mouse brain by magnetic resonance microscopy. Front. Neuroanat. 2:1 10.3389/neuro.05.001.2008 18958199 PMC2525925

[B24] MacKenzie-Graham A. Jones E. S. Shattuck D. W. Dinov I. D. Bota M. Toga A. W. (2003). The Informatics of a C57BL/6J mouse brain atlas. Neuroinformatics 1, 397–410 10.1385/NI:1:4:397 15043223

[B25] MacKenzie-Graham A. Lee E. F. Dinov I. D. Bota M. Shattuck D. W. Ruffins S. (2004). A multimodal, multidimensional atlas of the C57BL/6J mouse brain. J. Anat. 204, 93–102 10.1111/j.1469-7580.2004.00264.x 15032916 PMC1571243

[B26] Martone M. E. Gupta A. Ellisman M. H. (2004). E-neuroscience: challenges and triumphs in integrating distributed data from molecules to brains. Nat. Neurosci. 7, 467–472 10.1038/nn1229 15114360

[B27] Matthews D. W. (2012). The Architecture of the Mouse Trigeminal-Facial Brainstem?: Disynaptic Circuitry, Genomic Organization, and Follicle Mechanics. Ph.D. Dissertation, UC San Diego: b7625979.

[B28] Ng L. Bernard A. Lau C. Overly C. C. Dong H. W. Kuan C. (2009). An anatomic gene expression atlas of the adult mouse brain. Nat. Neurosci. 12, 356–362 10.1038/nn.2281 19219037

[B29] Orloff D. N. Iwasa J. H. Martone M. E. Ellisman M. H. Kane C. M. (2013). The cell: an image library-CCDB: a curated repository of microscopy data. Nucleic Acids Res. 41, D1241–D1250 10.1093/nar/gks1257 23203874 PMC3531121

[B30] Papp E. A. Kjonigsen L. J. Lillehaug S. Johnson G. A. Witter M. P. Leergaard T. B. (2013). Volumetric Waxholm Space atlas of the rat brain for spatial integration of experimental image data. Front. Neuroinform. Conference Abstract: Neuroinformatics 2013. 10.3389/conf.fninf.2013.09.00003

[B31] Paxinos G. (2004). The Mouse Brain in Stereotaxic Coordinates. Amsterdam: Gulf Professional Publishing

[B32] Paxinos G. Franklin K. B. J. (2001). The Mouse Brain in Stereotaxic Coordinates (Deluxe Edition), 2nd Edn. San Diego, CA: Academic Press

[B33] Paxinos G. Halliday G. Watson C. Koutcherov Y. Wang H. (2007). Atlas of the Developing Mouse Brain. San Diego, CA: Academic Press

[B34] Paxinos G. Watson C. (1998). The Rat Brain in Stereotaxic Coordinates. 4th Edn San Diego, CA: Academic Press

[B35] Paxinos G. Watson C. (2009). Chemoarchitectonic Atlas of the Mouse Brain. San Diego, CA: Academic Press

[B36] Portele C. (2007). OpenGIS Geography Markup Language (GML) Encoding Standard. Wayland, MA: Open Geospatial Consortium Rep. No. 3.2 1.

[B37] Ruffins S. W. Lee D. Larson S. D. Zaslavsky I. Ng L. Toga A. W. (2010). MBAT at the Confluence of Waxholm Space. Front. Neurosci. Conference Abstract: Neuroinformatics 2010. 10.3389/conf.fnins.2010.13.00132

[B38] Spellman P. Miller M. Stewart J. Troup C. Sarkans U. Chervitz S. (2002). Design and implementation of microarray gene expression markup language (MAGE-ML). Genome Biol. 3, research0046.1–research0046.9 10.1186/gb-2002-3-9-research0046 12225585 PMC126871

[B39] Susaki E. Tainaka K. Perrin D. Kishino F. Tawara T. Watanabe T. (2014). Whole-brain imaging with single-cell resolution using chemical cocktails and computational analysis. Cell 157, 726–739 10.1016/j.cell.2014.03.042 24746791

[B40] Swanson L. (1998). Brain Maps: Structure of the Rat Brain, 2nd Edn. Amsterdam: Elsevier

[B41] Toga A. W. (2002). Neuroimage databases: the good, the bad and the ugly. Nat. Rev. Neurosci. 3, 302–309 10.1038/nrn782 11967560

[B42] Van den Brink L. Portele C. Vretanos P. A. (2012). OpenGIS Implementation Standard Profile 10-100r3: Geography Markup Language (GML) simple features profile (with Corrigendum), in Technical Report (Open Geospatial Consortium Inc.). Available online at: http://www.opengeospatial.org/standards/gml (Accessed January 20, 2014).

[B46] West J. (2007). The economic realities of open standards: black, white and many shades of gray, in Standards and Public Policy, eds Greenstein S. Stango V. (Cambridge: Cambridge University Press), 87–122

[B43] Zakiewicz I. M. van Dongen Y. C. Leergaard T. B. Bjaalie J. G. (2011). Workflow and atlas system for brain-wide mapping of axonal connectivity in rat. PLoS One 6:e22669 10.1371/journal.pone.0022669 21829640 PMC3148247

[B44] Zaslavsky I. He H. Tran J. Martone M. E. Gupta A. (2004). Integrating brain data spatially: spatial data infrastructure and atlas environment for online federation and analysis of brain images, in Proceedings. 15th International Workshop on Database and Expert Systems Applications, 2004 (Zaragoza), 389–393 10.1109/DEXA.2004.1333505

